# SIRT1 Is a Potential Drug Target for Treatment of Diabetic Kidney Disease

**DOI:** 10.3389/fendo.2018.00624

**Published:** 2018-10-17

**Authors:** Yifei Zhong, Kyung Lee, John Cijiang He

**Affiliations:** ^1^Division of Nephrology, Longhua Hospital, Shanghai University of Traditional Chinese Medicine, Shanghai, China; ^2^Division of Nephrology, Department of Medicine, Icahn School of Medicine at Mount Sinai, New York City, NY, United States; ^3^Renal Section, James J. Peters VA Medical Center, Bronx, NY, United States

**Keywords:** SIRT1, acetylation, diabetic kidney disease, bromodomain inhibitor, podocytes

## Abstract

Multiple studies have demonstrated a critical role of Sirtuin-1 (SIRT1) deacetylase in protecting kidney cells from cellular stresses. A protective role of SIRT1 has been reported in both podocytes and renal tubular cells in multiple kidney disease settings, including diabetic kidney disease (DKD). We and others have shown that SIRT1 exerts renoprotective effects in DKD in part through the deacetylation of transcription factors involved in the disease pathogenesis, such as p53, FOXO, RelA/p65NF-κB, STAT3, and PGC1α/PPARγ. Recently we showed that the podocyte-specific overexpression of SIRT1 attenuated proteinuria and kidney injury in an experimental model of DKD, further confirming SIRT1 as a potential target to treat kidney disease. Known agonists of SIRT1 such as resveratrol diminished diabetic kidney injury in several animal models. Similarly, we also showed that puerarin, a Chinese herbal medicine compound, activates SIRT1 to provide renoprotection in mouse models of DKD. However, as these are non-specific SIRT1 agonists, we recently developed a more specific and potent SIRT1 agonist (BF175) that significantly attenuated diabetic kidney injury in type 1 diabetic OVE26 mice. We also previously reported that MS417, a bromodomain inhibitor that disrupts the interaction between the acetyl-residues of NF-κB and bromodomain-containing protein 4 (BRD4) also attenuates DKD. These results suggest that SIRT1 agonists and bromodomain inhibitors could be potential new therapuetic treatments against DKD progression.

## Introduction

Sirtuin family of nicotinamide adenine dinucleotide (NAD^+^)-dependent deacetylases, a homolog of yeast Sir2 (silent mating type information regulation 2), has been shown to play an important role in a variety of cellular functions. The mammalian Sir2 ortholog, Sirtuin-1 (SIRT1) is upregulated by caloric restriction and mediates the longevity effect of calorie restriction by regulation of glucose and lipid metabolism (1, 2). At the cellular level, SIRT1 regulates variety of processes including autophagy (3), energetic homeostasis (2), mitochondrial biogenesis (4), and apoptosis (5). A large body of evidence suggests that SIRT1 plays a major role in various kidney diseases by providing protection against cellular stresses associated with kidney injury (6–8). Here, we provide an overview of the role of SIRT1 in kidney cells in the context of diabetic kidney disease (DKD), with a focus on its role on the regulation of transcription factor activation. The review also discusses the potential new therapies by targeting SIRT1 pathway for DKD.

## Role of SIRT1 in regulation of transcription factor acetylation

Recent evidence suggests that transcription factor activation is regulated not only by protein phosphorylation, but also by acetylation. SIRT1 exerts biological effects not only through deacetylation of histones, but also deacetylation of various transcription factors that include p53, FOXO, RelA/p65, STAT3, PGC1α, and PPAR-γ (9), thereby leading to transcription repression. SIRT1 regulates p53 activity through deacetylation (10–13) and promotes cell survival through suppression of p53-dependent apoptosis in response to DNA damage and oxidative stress (5). SIRT1 was also shown to regulate the activities of FOXO family of transcription factors through deacetylation (14). Deacetylation of FOXO3 by SIRT1 enhances its ability to induce cell cycle arrest and resistance to oxidative stress, while inhibiting its ability to induce cell death (14, 15). We also showed that SIRT1 inhibits podocyte apoptosis through deacetylation of FOXO4 (16, 17). Several studies suggest that the transcriptional activity of signal transducer and activator of transcription 3 (STAT3) is also negatively regulated by SIRT1 (18–20). SIRT1 was found to cause the deacetylation and inactivation of STAT3 during caloric restriction (21). SIRT1 also exerts anti-inflammatory effects through the inhibition of NF-κB pathway. It was shown that the duration of nuclear NF-κB action is highly regulated by reversible acetylation (22, 23), and that SIRT1 inhibits the NF-κB signaling pathway through deacetylation of p65 (24). In addition, SIRT1 modulates cellular response to hypoxia via deacetylation of hypoxia-induced factor 1α (HIF-1α) (25–27). All these highlight an important transcription modulatory function by SIRT1 activity.

## SIRT1 provides renoprotection against DKD

Diabetic kidney disease (DKD) is the leading cause of chronic kidney disease and end-stage renal failure in the US (28). Even with optimal therapy, the incidence of DKD remains high, as none of the currently available therapy can reverse or completely forestall the progression of DKD. Therefore, development of more effective treatment for DKD is urgently required. The important role of SIRT1 in DKD has been demonstrated by number of studies (Table 1). We have previous shown that SIRT1 expression is significantly reduced in human kidney with DKD and that its reduction is more pronounced in the glomerular compartment than in the tubular compartment (17). An association between single nucleotide polymorphisms within *SIRT1* gene and DKD was observed in Japanese subjects with type 2 diabetes (40). However, the exact mechanism of regulation of SIRT1 expression in DKD remains unclear. On the cellular level, SIRT1 has been shown to regulate autophagy (41, 42) and oxidative stress response in the diabetic kidneys (35). Resveratrol was shown to attenuate DKD through activation of AMPK/SIRT1 pathway (29, 31) and by modulating angiogenesis (43). Studies have demonstrated a clear role of SIRT1 in renal tubular cells in the setting of acute kidney injury (6, 44). In diabetic kidneys, it was shown that reduced proximal renal tubular SIRT1 expression contributes to albuminuria by upregulation of the tight junction protein Claudin-1 in podocytes (32). Interestingly, reduction in *Sirt1* expression in tubular cells induced hypomethylation of the *claudin-1* gene in podocytes to promote its expression, while overexpression of *Sirt1* in tubular cells induced hypermethylation of *claudin-1* and downregulated expression in podocytes, indicating an important cross-talk between the two cell types and epigenetic regulation of Claudin-1 expression by SIRT1. Work from our laboratory also demonstrated a critical role of SIRT1 in podocyte injury in DKD. We showed that either knockdown or knockout of *Sirt1* specifically in podocytes aggravated DKD injury in type 2 diabetic *db/db* mice (33) and in STZ-induced diabetic mice (34). Importantly, our recent study demonstrated that the podocyte-specific overexpression of SIRT1 was sufficient to significantly attenuate podocyte injury and to impede DKD progression in type1 diabetic OVE26 mice. Together, these studies clearly demonstrate a protective role of SIRT1 against DKD in experimental models of both type 1 and type 2 diabetes.

**Table 1:** Summary of the *in vivo* studies of SIRT1 in DKD.

**Approaches**	**Animal models**	**Tissue/Cell types**	**Mechanisms regulated**	**References**
Dietary restriction	Diabetic Wistar fatty (fa/fa) rats	Whole kidney	Inflammatory; autophagy	(29)
Resveratrol	db/db mice	Whole kidney	Oxidative stress	(30)
Resveratrol	db/db mice	Whole kidney	AMPK/PGC1a; Oxidative stress	(31)
nicotinamide mononucleotide	STZ-induced diabetic and db/db mice	Tubule/podocyte crosstalk	Epigenetics, Claudin-1	(32)
Sirt1 knockout	Db/db mice	Podocytes	Inflammation; apoptosis	(33)
Pyridoxamine				
Sirt1 knockdown	STZ-induced diabetic mice	Podocytes	Mitochondria; senescence	(34)
hnRNP F	db/db mice	Tubular cells	Oxidative stress	(35)
Glycyrrhizic Acid	db/db mice	Whole kidney	AMPK/PGC-1a	(36)
Puerarin	STZ-induced diabetic eNOS-null mice	Podocytes	Oxidative stress; inflammation	(37)
Tangshen formula	STZ-induced diabetic rats	Whole kidney	NF-kB/inflammation	(38)
Sirt1 overexpression; agonists	OVE26 mice	Podocytes	Mitochondrial function; apoptosis	(39)

## Renoprotective mechanisms of SIRT1 in DKD

As the cellular and molecular mechanisms of SIRT1 has been recently reviewed (42, 45, 46), as well as the role and mechanism of other sirtuins in kidney disease (47), this review is focused primarily on its modulation of transcription factor through deacetylation in the setting of DKD.

### Effects of SIRT1 in inflammation in diabetic kidneys through NF-κB and STAT3 deacetylation

Many studies suggest that SIRT1 regulates activity of several transcription factors that regulate kidney cell homeostasis and are involved in pathogenesis of DKD through deacetylation. Systems biology analysis of microarray data suggests that JAK-STAT and NF-κB are key inflammatory pathways activated in diabetic kidneys (48, 49). Recently, we showed that the acetylation of STAT3 and RelA/p65 is increased in kidneys from diabetic patients and mouse models (33). More importantly, we demonstrated that the podocyte-specific knockout of *Sirt1* in *db/db* mice led to higher levels of p65 and STAT3 acetylation and resulted in greater degree of proteinuria and kidney injury than in control *db/db* mice, implicating SIRT1 as a key inhibitor of the NF-κB- and STAT3-induced inflammatory responses in DKD (33). In addition, we found that expression of the key pro-inflammatory factors mediated by NF-kB and Stat3 were also increased in the kidney of Sirt1 knockout *db/db* mice, further confirming a key role of Sirt1 in regulation of inflammation in the diabetic kidney.

### Effects of SIRT1 in cell death in diabetic kidneys through p53 and FOXO4 deacetylation

Several lines of evidence indicate that p53 mediates apoptosis of both podocytes and tubular epithelial cells in DKD (50–52). SIRT1 has been shown to promote cell survival by suppressing p53-dependent apoptosis in response to DNA damage and oxidative stress (5). The interplay of SIRT1-p53 pathway also controls cellular senescence (53–55). We reported previously that advanced glycation endproducts (AGEs) induce podocyte apoptosis through FOXO4-mediated Bim expression and that acetylation of FOXO4 is critical for mediating this effect (17). Overexpression of SIRT1 inhibited AGE-induced FOXO4 acetylation and podocyte apoptosis.

### Effects of SIRT1 in mitochondrial dysfunction and fibrosis in diabetic kidneys through of PGC-1α and smad3 deacetylation

SIRT1 has also been shown to regulate PGC-1α activity and to play an important role for maintenance of mitochondrial function in podocytes (56). The PGC-1α in regulation of mitochondrial function has been well described for neurodegenerative disorders (57). Both mitochondrial injury and cellular senescence are key pathological processes mediating kidney injury (58–60). Consistent with this, we have shown recently SIRT1 deficiency in podocytes aggravates aging-related kidney disease through enhanced cells senescence and mitochondrial dysfunction (61). Although the effects of SIRT1 on Smad3 acetylation remain to be determined, resveratrol was shown to affect acetylation but not phosphorylation of Smad3 to inhibit TGF-β1-induced up-regulation of collagen IV and fibronectin mRNA levels *in vitro* and renal fibrosis in the model of unilateral ureteral obstruction (UUO) *in vivo* (62). Therefore, it is plausible that increased SIRT1 activity may also attenuate renal fibrosis in DKD. Taken together, these studies suggest that SIRT1, as a negative regulator of inflammation, cellular senescence and mitochondrial dysfunction, is a key repressor of DKD pathogenesis.

## SIRT1 is a potential drug target for treatment of DKD

Given that SIRT1 is a key mediator in thwarting the progression of DKD and other kidney diseases, development of therapeutic strategies to specifically restore SIRT1 activity is warranted. In support of this, we recently demonstrated that increased SIRT1 expression in podocytes attenuated albuminuria and glomerular injury in OVE26 diabetic mice (39). As SIRT1 expression is reduced in diseased kidneys, identifying the molecular basis of its suppression in diabetic kidneys and to interfere in this process may be an avenue of therapeutic approach. We previously showed that increased advanced glycation endproducts (AGEs) in in the diabetic milieu contribute to reduced SIRT1 expression in podocytes (17). Inhibition of AGE formation by pyridoxamine *in vivo* restored SIRT1 expression the glomeruli of *db/db* mice and attenuated podocyte injury and progression of DKD (17). Similar observations of SIRT1 reduction by AGEs were made in mesangial cells *in vitro* (63).

Another approach would be to stimulate SIRT1 activity through SIRT1 agonists. Resveratrol is a well-known SIRT1 agonist that has shown to improve DKD in several animal models (30, 31). However, recent reports indicate that resveratrol may not be a SIRT1-specific (64), nor are other purported SIRT1 agonists, such as SRT1720, SRT2183, and SRT1460 (65). We also showed that puerarin, an extract from a Chinese herbal medicine, attenuates diabetic kidney injury through activation of SIRT1 and suppression of NOX4 expression in podocytes in experimental diabetic mouse model (62). Other herbal medicines or compounds have also been reported to improve DKD through activation of SIRT1 (36, 38). Metformin is reported to improve podocyte function by activating SIRT1 (66). However, SIRT1-independent effects on podocytes or in DKD by non-specific SIRT1 agonists or metformin cannot be ruled out. Recently, we developed a new potent and selective SIRT1 agonist, BF175 (39). In cultured podocytes BF175 increased SIRT1-mediated activation of PGC1-α and protected against high glucose-mediated mitochondrial injury. *In vivo*, administration of BF175 for 6 weeks in type 1 diabetic OVE26 mice resulted in a marked reduction in albuminuria and in glomerular injury in a manner similar to podocyte-specific SIRT1 overexpression. BT175 treatment also attenuated diabetes-induced podocyte loss and reduced oxidative stress in glomeruli of OVE26 mice. Therefore, BT175 and its analogs could be developed as novel therapeutic strategy to treat DKD. However, these approaches of targeting SIRT1 are not without limitations. As discussed above, the specificity of the SIRT1 agonists remains a concern. Given the heterogeneity of SIRT1 function, the ever expanding list of its substrates, and the different effects of deacetylation on its target protein functions, it's possible that the beneficial effects of SIRT1 are mixed with potentially pernicious side effects.

As SIRT1 exerts its renoprotective effects through deacetylation of key transcription factors (TFs) involved in DKD, another therapeutic approach may be to directly regulate the transcription factor acetylation through bromodomain inhibitors (BrDi). Acetylated lysines of the key TFs involved in DKD pathogenesis such as p65 NF-κB interact with proteins containing bromodomains (67), and BrDi could suppress their acetylation in a more specific manner. For instance, NF-κB transcriptional activity is dependent upon its acetylation at lysine 310 (Lys310), and Lys310-acetylated p65 NF-κB recruits the BET protein BRD4 in complex with positive transcription elongation factor b (p-TEFb) and RNA polymerase II that together form a productive transcriptional machinery complex (68). We reported that a BET-specific BrDi MS417 suppresses TNF-α-induced acetylation of p65 NF-κB and the expression of NF-κB target genes in kidney cells *in vitro* and attenuates proteinuria and glomerulosclerosis in a mouse model of HIV-associated nephropathy *in vivo*. MS417 also inhibited AGE-induced acetylation of p65 NF-κB in podocytes *in vitro* and mitigated proteinuria in diabetic *db/db* mice. Therefore, MS417 or other BrDi might be another class of potential drug candidates to treat DKD patients.

**Figure 1:**
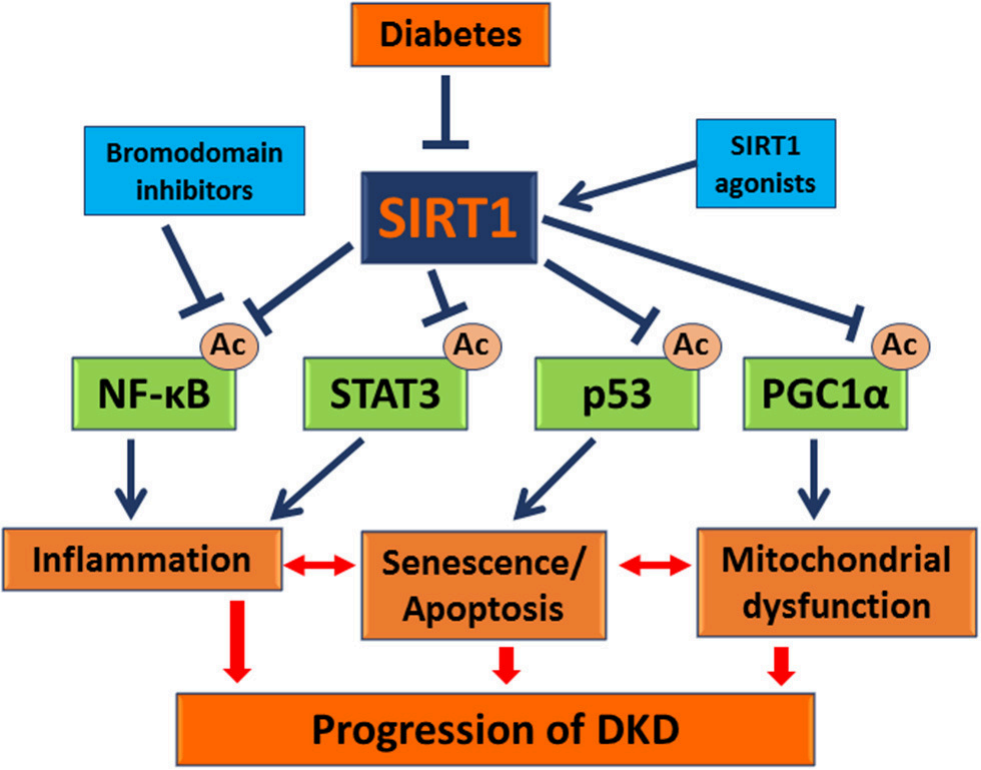
Role of SIRT1 in DKD pathogenesis. This schema summarizes how Sirt1 mediates podocyte injury in DKD. The data suggest that Sirt1 expression is reduced in the diabetic glomeruli including podocytes. Reduced SIRT1 expression leads to increased acetylation and activation of transcription factors, such as NF-κB, STAT3, p53, and PGC1α, leading to exacerbated inflammation, senescence/apoptosis, and mitochondrial dysfunction of kidney cells such as podocytes (shown in blue arrows). All these processes interact each other and contribute to the progression of DKD (shown in red arrows). Therefore, SIRT1 agonists or inhibition of transcription factor acetylation through use of bromodomain inhibitors will reverse these diseased processes and could be developed to treat DKD. (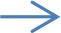): stimulation; (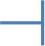): inhibition; (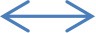): interaction.

In summary, SIRT1 has significant renoprotective effects against podocyte injury in DKD (Figure 1), and SIRT1 agonists and bromodomain inhibitors are promising candidates as therapeutic approach in treatment of DKD patients.

### Clinical perspectives

Since a large amount of evidence suggest that Sirt1 is a key molecule involving in the pathogenesis of DKD and the expression of Sirt1 is suppressed in human diabetic kidney, enhancing the SIRT1-induced transcription factor deacetylation via SIRT1 agonists or bromodomain inhibitors may serve as potential therapies for human DKD.

## Author contributions

All authors listed have made a substantial, direct and intellectual contribution to the work, and approved it for publication.

### Conflict of interest statement

The authors declare that the research was conducted in the absence of any commercial or financial relationships that could be construed as a potential conflict of interest.
